# Antioxidant Enzyme Activity and Serum HSP70 Concentrations in Relation to Insulin Resistance and Lipid Profile in Lean and Overweight Young Men

**DOI:** 10.3390/antiox12030655

**Published:** 2023-03-06

**Authors:** Anna Lubkowska, Wioleta Dudzińska, Waldemar Pluta

**Affiliations:** 1 Department of Functional Diagnostics and Physical Medicine, Pomeranian Medical University in Szczecin, Żołnierska 54, 71-210 Szczecin, Poland; 2 Department of Physiology and Biochemistry, Institute of Biology, University of Szczecin, Felczaka 3c, 71-412 Szczecin, Poland

**Keywords:** antioxidant enzyme activity, HSP70, insulin resistance

## Abstract

Oxidants are generated by all cells during normal oxidative respiration, and as long as they are under the control of appropriate mechanisms, they act as intracellular signaling molecules participating in complex functions. Oxidative stress can also affect insulin levels in the body. The production of reactive oxygen species by-products can lead to insulin resistance. Heat shock proteins (70 kDa) protect cells from the damaging effects of heat shock but also oxidative stress. The aim of the study was to investigate the serum concentration of HSP70 in young, non-obese but overweight men (BMI ≤ 30 kg/m^2^) and to assess its association with the insulin resistance, lipid profile and antioxidant system of red blood cells. Fifty-seven young men were examined and divided into two groups: lean men (n = 30) and men overweight (n = 27). A statistically significant difference was observed in the BMI (*p* < 0.007), HSP70 concentration (*p* < 0.000), serum insulin concentration (*p* < 0.000), HOMA-IR (*p* < 0.0001), superoxide dismutase (*p* < 0.02) and glutathione peroxidase (*p* < 0.05) between the studied groups. There was a negative correlation between the concentration of HSP70 with the insulin level (r = −0.50; *p* < 0.0004) and with the HOMA-IR (r = −0.50; *p* < 0.0004). These changes were associated with an increase in the activity of antioxidant enzymes. Our findings suggest that measuring the extracellular concentration of HSP70 can be an important indicator in disorders of glucose homeostasis.

## 1. Introduction

Insulin resistance associated with chronic inflammatory conditions result in increased oxidative stress, modification of cellular proteins and a reduced cellular defense system [1]. Oxidative stress is defined as an imbalance in the redox status due to the excessive production of reactive oxygen species (ROS), which include both the free radicals and their non-radical intermediates. The first of these are defined as forms containing one or more unpaired electrons. This incomplete electron shell confers their high reactivity. Although free radicals can be produced from many elements, the most important in the biological system are those formed with the participation of oxygen and nitrogen. The most common free radical under physiological conditions is the superoxide anion (O2^•−^), generated mainly by mitochondria. Superoxide detoxification requires the superoxide dismutase enzyme (SOD), which converts O2^•−^ to hydrogen peroxide (H_2_O_2_). This one, in turn, is detoxified into water by catalase (CAT) and glutathione peroxidase (GPx). Important in antioxidant enzyme activity is cooperation, because an imbalance in the O2^•−^ and H_2_O_2_ concentrations can result in the formation of superoxide anion hydroxyl ion (OH^•^), which is much more dangerous [2].

Oxidants are generated by all cells during normal oxidative respiration. Under strict regulation, ROS act as intracellular signaling molecules, contributing to complex functions such as blood pressure regulation, cognition and the immune response [3]. On the other hand, their excess disrupts many important cellular processes and functions, including cell proliferation and differentiation, inflammation or fatty acid peroxidation. Moreover, they can cause oxidative damage to DNA, proteins and membrane lipids. Persistent oxidative stress can lead to health disorders, including diabetes, neurogenerative syndromes and even cancer [4].

The relationship between oxidative stress and insulin resistance has long been recognized. Research in this area has shown a strong correlation between the body’s oxidative stress level and the presence of insulin resistance. The impairment of insulin sensitivity may be caused by hyperglycemia and hyperlipidemia-induced ROS or the production of ROS by-products. As a result, structural and functional changes of the insulin molecule occur, ultimately leading to a reduction in its bioactivity and activation of redox-sensitive cell signaling pathways, which interfere with insulin, signaling and the transport of glucose cells [3].

Heat shock proteins 70 kDa (HSP70) are the most ubiquitous molecular chaperones in the human body, which are located in all the cellular compartments. The scope of activities of HSP70 is very broad and covers the folding of both nascent and misfolded proteins, protein assembly, the regulation of their activity and the degradation of and prevention from dismantling protein aggregates [5]. In this way, HSP70 protects cells not only from the damaging effects of heat shock but also oxidative stress (OS) [6]. In addition, HSP70 are very good therapeutic target for viral infections, as we have described in our previous work [7]. This multifaceted action makes HSP70 an interesting research target. Experimental evidence shows that there is a correlation between HSP70 and the redox status. On the one hand, both oxidative stress and antioxidants appear to regulate HSP70 expression [8,9]. On the other hand, reducing the HSP70 expression may increase reactive oxygen species production and the oxidation of mitochondrial proteins [10]. Moreover, HSP70 reduces cellular damage caused by oxidative stress in kidney cells [11] and increases the activity of glutathione peroxidase and glutathione reductase (GR) in response to hypoxic and ischemia [12].

Data on the concentration of HSP70 in lean and overweight Caucasian subjects and its possible association with the insulin resistance, lipid profile and antioxidant system of red blood cells are insufficient. Against this background, our aim was to investigate the serum concentration of HSP70 in young, non-obese men (body mass index (BMI) ≤ 30 kg/m^2^) and to assess its association with the insulin resistance, lipid profile and antioxidant system of red blood cells.

## 2. Materials and Methods

### 2.1. Participants

The study involved 57 healthy men, without diabetes, aged 22 to 26 years and taking no medications. All subjects were students of the General Tadeusz Kościuszko Military Academy of Land Forces. Volunteers were divided into two groups: lean and non-obese but overweight, according to their body mass index (BMI). According to the WHO criteria for Western populations, a slim person is defined by a BMI of 18.5–24.9 kg/m^2^, and a person overweight by a BMI of 25–29.9 kg/m^2^. The groups were homogeneous in terms of diet and daily physical activity, due to the scheduled joint activities, accommodations and food provided by the Military Academy. The exclusion criteria for participation in the study was: metabolic diseases, diabetes, hypertension, and BMI < 18.5 or ≥30 kg/m^2^. According to the Declaration of Helsinki, each participant gave written consent before participating in the study. The local ethics committee approved the study (Pomeranian Medical University; Ref. KB-0012/54/10).

### 2.2. Measures

#### 2.2.1. Biochemical Parameters of Venous Blood

Venous blood was drawn by qualified medical personnel from each of the volunteers after overnight fasting between 6.00 a.m. and 8.00 a.m. after a 10 min rest in a sitting position from the antecubital vein using Vacutainer tubes (Sarstedt, Nümbrecht, Germany) and separated into two tubes: one for biochemical analysis of the serum (4.9 mL) and the other to determine the blood counts (1.2 mL anticoagulated with 1 g/L K2 EDTA). 

The HSP-70 protein levels were determined using commercially available enzyme-linked immunosorbent assay (ELISA) kits (EIAab, Wuhan, China). The standard curve had a range of 0.2–10 ng/mL, the intra-assay: <8% and inter-assay: <10%. The serum levels of apolipoprotein A (ApoA) and apolipoprotein B (ApoB) were determined using commercially available ELISA kits (EIAab, Wuhan, China), according to the instructions given by the manufacturer. The detection limits for the lipoprotein tests were 0.8 and 892.9 µmol/L, the intra-assay: <8% and inter-assay: <10%. The serum level of interleukin 3 (Il-3) was determined using commercially available ELISA kits from R&D Systems (Abingdon, UK), according to the instructions given by the manufacturer. The detection limits for the interleukin 3 test were 31.2–2000 pg/mL, the intra-assay: <8% and inter-assay: <10%. The insulin levels were determined by the ELISA method using reagent kits (DRG Medtek, Warsaw, Poland). The detection limit for insulin was 1 mU/L. The insulin resistance IR (HOMA-IR) was calculated according to the formula: fasting plasma glucose (mmol/L) × fasting plasma glucose (mmol/L)/22.5.

To determine the extracellular hemoglobin, haptoglobin, total bilirubin, total protein, albumin, uric acid, glucose, total cholesterol, HDL cholesterol and triglycerides, a spectrophotometric method was used. The concentration of the LDL cholesterol fraction was determined by a direct method. After the determinations, the obtained lipid profile was supplemented by the calculation of the TG:TCh, TCh:HDL and LDL:HDL ratios. 

The GSH_total,_ GSH_reduced,_ GSSG and GST concentrations in the hemolysate samples were determined by the colorimetric method (OxisResearch, Portland, OR, USA). SOD, CAT, GPx and GSSG-R activity were also measured with a BIOXYTECHH kit (OxisResearch, Portland, OR, USA) using a UV/VIS Lambda 40 (Perkin-Elmer, Wellesley, MA, USA) spectrophotometer. SOD: sensitivity: 0.1 U/mL, specificity: 97% and coefficient of variation: lower than 4%; CAT: sensitivity: 1.71 U/mL, specificity: 89% and coefficient of variation: lower than 2%; GPx: sensitivity: 6 U/L, specificity: 94% and coefficient of variation: lower than 4%, GSSG-R: sensivity:0.14 U/L, specificity: 94% and coefficient of variation: lower than 4% and GSH/GSSG: sensitivity: 5 mmol/L, specificity: 95% and coefficient of variation: lower than 2%. The enzyme activity and glutathione concentration were calculated per 1 g of erythrocyte hemoglobin. After the determinations, the GSH:GSSG ratio was calculated.

#### 2.2.2. Statistical Analysis

To determine the sample size necessary to assure appropriate statistical precision, the G*Power 3.1.9.4 program was used. The established effect size was 0.8, α error probability was 0.05 and power was 0.8. The non-central parameter δ was 2.88. The total sample size was 52 and actual power 0.807. The obtained results were statistically analyzed using MS Excel and Statistica 13.3 software (Statistica PL, StatSoft, Kraków, Poland). The normality of the distribution was checked using the Shapiro-Wilk test. The test results showed that the distributions of the examined values deviated from the normal distribution; therefore, in detailed statistical analyses, non-parametric tests were used. The results were presented as the middle value of the distribution—median, lower quartile value (Q25) and upper quartile value (Q75).

In order to demonstrate the significance of the differences, the non-parametric ANOVA Kruskal–Wallis rank test and the Mann–Whitney *U* test were used. The significance level was assumed at *p* < 0.05. To prove whether the observed correlations were statistically significant, the Spearman’s rank correlation coefficient significance test was used.

## 3. Results

### 3.1. Anthropometrical and Biochemical Parameters in the Study Groups

**Figure 1:**
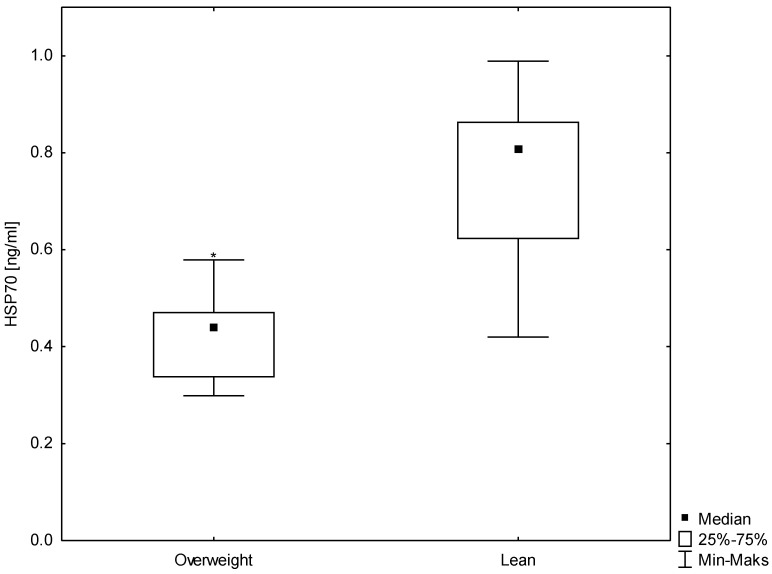
A box-whisker plot for the concentrations of HSP70 in the study groups. * statistically significant difference at *p* < 0.05.

**Figure 2:**
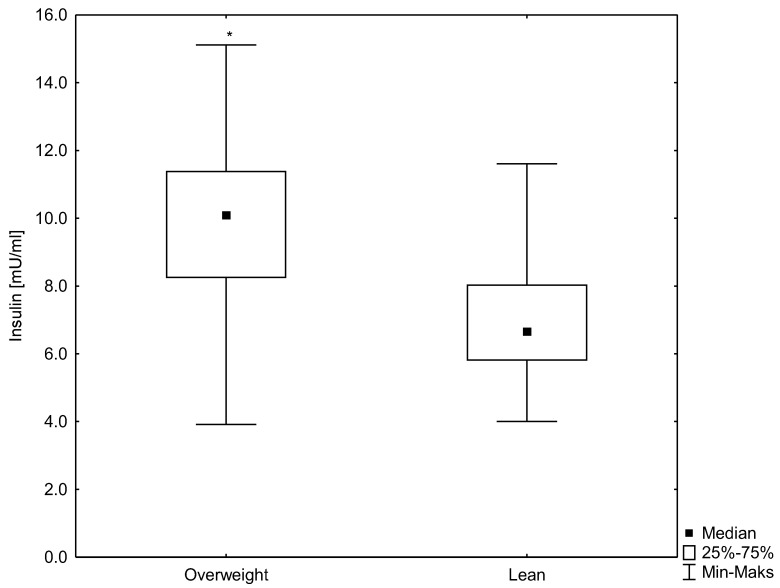
A box-whisker plot for the insulin concentrations in the studied groups. * statistically significant difference at *p* < 0.05.

**Figure 3:**
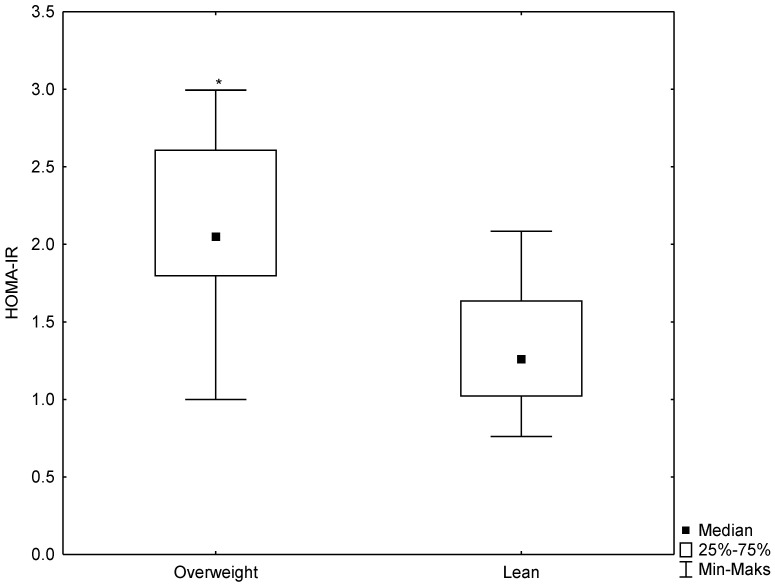
Box-whisker plot for HOMA-IR values in the study groups. * statistically significant difference at *p* < 0.05.

According to the WHO guidelines, the participants were divided into two groups: lean men (n = 30) and men overweight (n = 27). A statistically significant (*p* < 0.007) difference was observed in the BMI between the studied groups. Highly statistically significant (*p* < 0.000) differences were demonstrated in HSP70 concentrations (median 0.8 ng/mL for the lean group and 0.4 ng/mL for the group overweight) and in the serum insulin concentrations (6.7 mU/mL for the lean group and 10.0 mU/mL for the group overweight). Moreover, a difference was observed in the HOMA-IR, which was significantly higher in men overweight (median 1.3 mM for the lean group and 2.0 mM for the group overweight, *p* < 0.0001) (Figure 1, Figure 2 and Figure 3). No differences were found for the other biochemical parameters. These results are summarized in Table 1.

### 3.2. Erythrocyte Antioxidant Enzyme Activity and Glutathione Concentration in Study Groups

The analysis of antioxidant enzyme activity showed a statistically significant difference between the concentration of SOD (median 762 U/gHb for the lean group and 872 U/gHb for the group overweight, *p* < 0.02) and GPX (median 6.7 U/gHb for the lean group and 8.3 U/gHb for the group overweight, *p* < 0.05). No differences were found for other antioxidant enzyme activity. These results are summarized in Table 2.

### 3.3. Relationships between HSP70 Level and Concentration of Insulin

**Figure 4:**
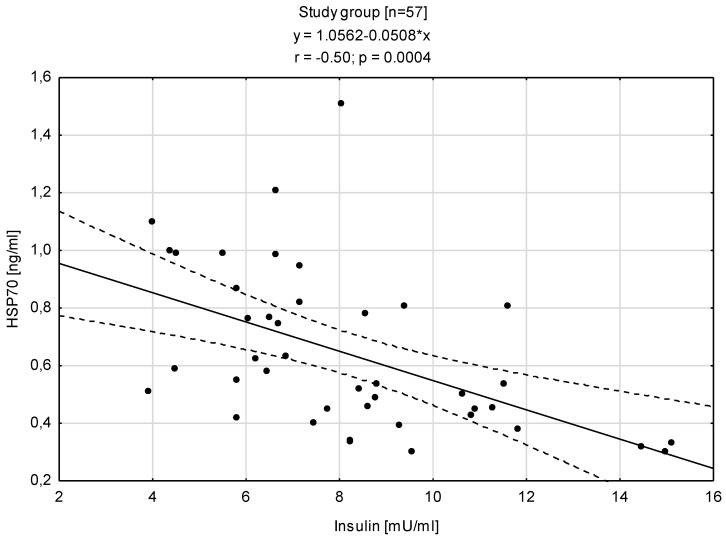
Relationship between the concentration of HSP70 and insulin concentration in the blood serum.

**Figure 5:**
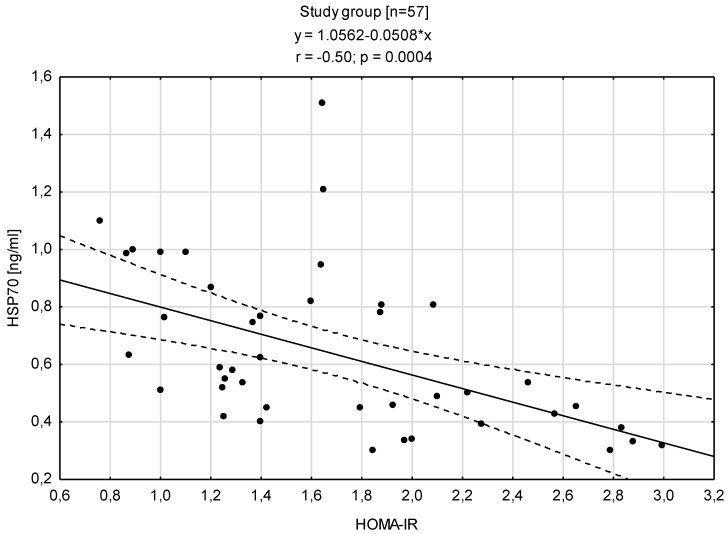
Relationship between the concentration of HSP70 in the blood serum and the HOMA-IR index.

Analyzing the relationships between HSP70 level and concentration of insulin showed that is a negative correlation with the insulin level (r = −0.50; *p* < 0.0004) and the HOMA-IR (r = −0.50; *p* < 0.0004) (Figure 4 and Figure 5).

## 4. Discussion

The conducted research was aimed at investigating the serum concentration of HSP70 in young, non-obese men (BMI ≤ 30 kg/m^2^) and to assess its association with the insulin resistance, lipid profile and antioxidant system of red blood cells. In our study, we observed a negative correlation between the HSP70 level and concentration of insulin (r = −0.50; *p* < 0.0004). The HSP70 concentration was higher in the lean group (median 0.8 ng/mL) compared to the group overweight (median 0.4 ng/mL) at the same time with a lower insulin concentration (median 6.7 vs. 10.0 mU/mL). This may prove the protective role of HSP70 against insulin resistance. The analysis of the activity of antioxidant enzymes showed a higher concentration of SOD in the group overweight (median 872 vs. 762 U/gHb) and GPX (8.3 vs. 6.7 U/gHb).

We have investigated the possible link among erythrocyte antioxidant enzyme activity (SOD, CAT, GPX, GST and GSSG-R); GSH_total_; GSH_reduced_ and GSSG concentrations with a GSH:GSSG ratio and the level of HSP70 in serum in two groups of young, physically active men, with differences in the BMIs between the groups (lean vs. overweight). 

According to our results, alterations of the antioxidant enzymes related to body mass are not uniform. The interesting finding from the present study is the lower erythrocyte activity of SOD and GPX, with an accompanying higher concentration of serum HSP70 in the group of men with a lower BMI (lean group). 

The human body’s defense mechanisms against oxidative stress are complex and involve cellular and extracellular antioxidant systems regulated at multiple levels. The cellular defense against ROS generated during oxidative metabolism utilizes antioxidant enzymes [13], including superoxide dismutase, catalase and glutathione peroxidase.

SOD catalyzes the dismutation of superoxide anion (O_2_^−^) to hydrogen peroxide (H_2_O_2_) in the first step of the defense mechanism, which is followed by CAT and GPX1 independently converting H_2_O_2_ to water. An increase in the SOD catalytic activity produces an excess of H_2_O_2_ that must be efficiently neutralized by either CAT or GPX1 (using GSH as a thiol donor); otherwise, H_2_O_2_ reacts with O_2_^−^ produced in the Haber–Weiss reaction hydroxyl radical OH, which is more dangerous [14]. The physiological role of GPXs is mediated by its involvement in the redox regulation of cellular functions.

Our results regarding higher SOD and GPx activity in overweight men are consistent with some of the literature data. However, it should be noted that the results of studies on the activity of SOD and GPX, depending on the nutritional status, are, in some cases, convergent but not in others. Vávrová et al. showed altered erythrocyte activities of antioxidant enzymes in patients with metabolic syndrome (MetS) with central obesity who had higher activities of SOD and GR than in healthy subjects, though the activity of GPX1 was not significantly changed [15]. Karaouzene et al. (2011) demonstrated that the SOD levels were differentially associated with obesity in young and old obese subjects. The response to oxidant damage differs according to age, because the maturation of antioxidant enzymes is related to aging [16]. Erdeve et al. evaluated the antioxidative Cu/Zn-SOD response to obesity-related stress in obese children and stated that a high-caloric diet may induce mitochondrial oxidative metabolism and cause electron leakage from a mitochondrial respiratory chain in the obese, which leads to an increase in the SOD level [17].

The altered expression and activity of the glutathione peroxidases have been observed along with obesity in human and animal studies. Mice with the elevated expression of a major antioxidant selenoprotein (GPX1) showed hyperglycemia, hyperinsulinemia, elevated body fat accretion and plasma leptin and reduced insulin sensitivity. It is postulated that the most plausible mechanism could be the effect of GPX1 overexpression on the intracellular H_2_O_2_ tone. Normal or minimal levels of intracellular ROS or H_2_O_2_ are required for sensitizing insulin signaling. The overexpression of GPX1 may accelerate the quenching of the intracellular H_2_O_2_ burst after insulin stimulation, resulting in less inhibition of the protein–tyrosine phosphatase activity and, subsequently, attenuated phosphorylation of the insulin receptor [18]. Rupérez et al. found that GPX activity was found to be positively and significantly correlated with blood pressure, adipocyte fatty acid-binding protein and high-sensitivity C-reactive protein. Moreover, the GPX variant GPX1-7 genes: rs757228, rs8103188, rs445870 and rs406113 were associated with prepubertal childhood obesity [19]. Chen et al. observed a significantly positive association between increases in erythrocyte GPX1 activity and levels of insulin resistance in normal pregnant women [20].

The erythrocyte and serum GPX activity were appropriately 26% and 22% higher in obese Brazilian women and the Central Mexican population compared to the controls, being associated with insulin sensitivity and the atherogenicity index. GPX activity was characterized by a considerable increase in obese and centrally obese diabetic subjects in parallel with oxidative stress markers [21,22,23]. The cellular response to oxidant damage may differ from cell to cell because of different quantities and activities of antioxidant enzymes. The antioxidant enzyme levels increase to prevent the destruction of tissues in a state of oxidant damage and also during compensatory adaptation to oxidative stress during the development of obesity [24]. 

Although, in our study, no association was found between the components of the antioxidant response and serum HSP70 level, it should be noted that significantly lower values for the 70 kDa heat shock proteins were recorded in the overweight group of men. 

We decided to analyze the serum HSP70 level according to the BMI and antioxidant activity, due to the fact that the studies carried out so far have shown a wide range of extracellular HSP70 activity both in triggering the signaling of the proinflammatory cascade and in blocking it (in the case of excessive activation of the immune system) [25,26,27].

Extracellular HSP70 is still a topic of interest for researchers who are looking for relationships between the protein concentration and the occurrence of diseases, inflammation and pathology, as well as in relation to the nutritional status and the aging process. Martínez de Toda and De la Fuente concluded that HSP70 plays a key protective role in the cell aging process. In addition, it can be a biomarker of the rate of aging and the lifespan [28]. The response of cells to stress involves the induction of the synthesis of HSP. A variety of cell types can release HSP70. It is suggested chaperone secretion takes place both in functioning and dying cells, and its impact involves various receptors [29]. The release of HSP70 from dying cells can be a signal of danger, while secretion from living cells is a signal of a proper response to stress. Extracellular HSP70, by inducing the release of proposal cytokines (e.g., TNFα, IL-6, IL-1b or Toll-like 2 receptors), can stimulate the HPA axis. This results in the increased secretion of both glucocorticoids and other adrenal steroids—powerful anti-inflammatory agents. Remarkably, glucocorticoids, at certain levels, can lead to an increase in IL-6 production. Therefore, it can be argued that the interactions of HSP70–glucocorticoid–IL-10 may be an important element of the anti-inflammatory mechanism [30]. Our results suggest that this mechanism may be less effective in people overweight. Chronic inflammation associated with overweightness and obesity can be compared to the oxidative–inflammatory theory of aging proposed by Fuente and Miquel [31]. They assumed that the chronic state of oxidative and inflammatory stress is the cause of age-related changes—in particular, those affecting the nervous, endocrine and immune systems. In the literature, you can find information on the decrease in HSP70 concentration with increasing age [32,33].

According to our results, Islam et al. demonstrated that the HSP70 concentration is inversely correlated with the BMI, percentage body fat, waist circumference and insulin resistance [34].

The proposed reason for the lower serum HSP70 levels in young men overweight could be a compromised expression of specific heat shock proteins such as HSP70, impaired synthesis of intracellular HSP and/or heat shock factor 1 (HSF-1)-dependent induction. It has been demonstrated that reduced activity of the anti-inflammatory HSP70 pathway correlates with nonalcoholic fatty liver disease (NAFLD) progression and with markers of oxidative stress in the obese patient, paralleled by similar reductions in HSF1 and insulin resistance [35]. 

On the other hand, HSP70 can be released from cells by an active mechanism that is independent of de novo HSP70 synthesis or cell death. Assuming the same amount of intracellular HSP70 synthesis, the active release mechanism by which HSP70 enters the circulation could be defective.

The mechanisms for the secretion of HSP70 are complex and incompletely understood. 

HSP70 release involves transit through an endolysosomal compartment and is inhibited by lysosomotropic compounds. Moreover, the rate of HSP70 secretion correlates with the appearance of the lysosomal marker LAMP1 (lysosome-associated membrane proteins) on the cell surface, further suggesting the role of endolysosomes in the extracellular ATP regulatory role [36]. Recent findings demonstrated that the disruption of lipid rafts on a cell membrane abrogates the release of HSP70 from living cells [37].

Our study also showed that the serum concentration of HSP70 in men overweight was significantly lower than in those with normal body weight and that the decrease in HSP70 concentration was accompanied by an increase in insulin resistance.

It is widely known that, in obesity, chronic low-grade inflammation and a disturbed balance between oxidative stress and the antioxidant defense system are significant in the inhibition of the insulin receptor signaling cascade and strongly associated with insulin resistance and type 2 diabetes [38]. There is also a known relationship between impairment of the heat shock response and diabetes and insulin resistance. In patients with type 2 diabetes, decreased HSP72 gene expression in muscles is correlated with decreased tissue insulin sensitivity [39,40]. These observations were also confirmed in cell and animal models [41,42]. A decreased expression of intracellular HSP was also linked with the metabolic syndrome, which is known to be proceeded by insulin resistance [43].

On the other hand, the authors of other studies showed that HSP72 expression in the subcutaneous adipose tissue in a diabetic obese group was reduced compared to nondiabetic obese subjects, whereas, in nondiabetic obese subjects, a significantly higher expression of this protein was observed compared to lean subjects [44]. Therefore, it seems that obesity without diabetes may trigger an increase in HSP expression in adipose tissue. On the contrary, Di Naso et al. [35] observed decreased levels of HSF1 / HSP70 in the liver and adipose tissue of obese patients in the course of NAFLD. Additionally, the serum concentration of HSP70 in obese patients with NAFLD turned out to be significantly lower compared to the non-obese control group [45].

These divergent results may be related both to different clinical profiles of patients and to different criteria for their inclusion, which makes the comparison difficult and imprecise.

Despite some experimental studies on intracellular HSP70 expression, there is very little data on measuring the serum HSP70 concentration and determining its association with insulin resistance. While the intracellular HSP levels are lowered in diabetes and correlated with insulin resistance, in many studies, the levels of extracellular HSP72 (serum/plasma) are elevated in type 1 and type 2 diabetes [46,47,48] and correlated with oxidative damage and stress [47], disease duration [46] and with the CRP levels, monocytes and TNF-α [49,50]. It has also been reported that the serum HSP72 levels are elevated in women with long-term diabetes compared to men and do not decrease after hypoglycaemic therapy in women with newly diagnosed diabetes, but they do decrease in men [46]. In one study on patients with type 1 diabetes, an increase in HSP72 was observed in diabetic ketoacidosis, which was significantly reduced after treatment [47]. In contrast, another study showed immeasurable levels of HSP70 in the serum of patients with type 1 diabetes, with and without microvascular complications [51]. Thus, again, the discrepancy in the results makes the underlying mechanisms for altering the extracellular concentration of HSP70 remain inconclusive. While it is still unclear how and from where HSP70 is released into the circulation, our study shows that the serum levels of HSP70 in men overweight without metabolic disease are significantly lower compared to men with a normal body weight and that there is a decrease in the HSP70 levels accompanied by an increase in insulin resistance. Our results are clearly supported by studies carried out in a group of healthy African American men and women [34], in which an increase in the BMI, percentage of adipose tissue, waist circumference and insulin resistance were accompanied by a significantly reduced concentration of HSP70 in the blood serum, which is correlated with insulin resistance.

Thus, our findings are consistent with the hypothesis that insulin resistance may contribute to the reduction of HSP70 levels [52,53]. Significantly lower HSP72 protein expression in skeletal muscle was associated with increased obesity and decreased insulin sensitivity in healthy subjects. The relationship between HSP72 protein expression and insulin sensitivity is explained by adiposity [54]. This hypothesis is consistent with the rodent data for which heat treatment and overexpression of HSP72 have been shown to protect against high-fat diet-induced insulin resistance. Heat treatment resulted in the decreased activation of Jun NH2-terminal kinase (JNK) and inhibitor of κB kinase (IKK-β), stress kinases implicated in insulin resistance and upregulation of HSP72 and HSP25, proteins previously shown to inhibit JNK and IKK-β activation, respectively [55]. Similarly, the induction of HSP72 and HSP27 by heat in human monocytes of obese individuals resulted in the dampening of IKK-β and JNK stress kinase activation and improved insulin signaling [56]. Although we only assessed statistical differences in the circulating HSP70 levels for overweight and insulin resistance, our findings are scientifically supported and warrant further investigation of possible mechanisms. The main limitation of this study is its cross-sectional nature, which makes it impossible to determine the direction of causality. Prospective studies are needed to confirm and improve the current results. Second, we had no data on participants’ eating and physical activity habits that could influence insulin resistance, overweight/obesity and the HSP70 protein.

## 5. Conclusions

To our knowledge, this study is the first to show a clear negative correlation between the insulin resistance and serum HSP70 concentration levels in men overweight. These changes were associated with an increase in the activity of antioxidant enzymes. These observations are noteworthy, because disturbances in the glucose homeostasis (decreased sensitivity of peripheral tissues to insulin) are an important predisposing factor for the development of many metabolic diseases in the future, including type 2 diabetes, metabolic syndrome and polycystic ovary syndrome. Hence, our findings suggest that measuring the extracellular concentration of HSP70 (which is relatively easy to assess) can be an important indicator under such conditions. More research is needed to identify causal relationships and elucidate the theme.

## Figures and Tables

**Table 1:** Anthropometrical and biochemical parameters in the study groups.

	Lean n = 30 Median (Q_25_–Q_75_)	Overweight n = 27 Median (Q_25_–Q_75_)	*p<*
Age (years)	23.0 (23–23)	23.5 (23–25)	NS
BMI (kg/m^2^)	23.6 (22.83–24.25)	26.9 (26.30–28.40)	0.007
Extracellular Hb (g/dL)	30.0 (26.53–37.07)	31.2 (28.48–39.99)	NS
Haptoglobin (g/L)	0.3 (0.10–0.37)	0.2 (0.14–0.32)	NS
Total bilirubin (mg/dL)	1.5 (1.15–1.69)	1.4 (1.27–1.44)	NS
Erythropoietin (ng/mL)	263 (210–320)	246 (200–278)	NS
Il-3 (pg/mL)	18.1 (10.14–28.17)	18.9 (13.78–35.29)	NS
Total protein (g/dL)	7.1 (6.66–7.54)	7.4 (6.87–7.43)	NS
Albumin (g/dL)	51.2 (47.53–54.38)	48.5 (42.90–50.86)	NS
Uric acid (mg/dL)	5.0 (4.43–6.13)	5.8 (4.71–7.01)	NS
Glucose (mM)	4.8 (4.30–5.15)	5.3 (4.33–5.52)	NS
Total cholesterol (mg/dL)	196 (154–216)	188 (163–211)	NS
HDL cholesterol (mg/dL)	25.3 (24.25–28.14)	23.9 (22.44–27.72)	NS
LDL cholesterol (mg/dL)	142 (103–158)	135 (112–162)	NS
Triglycerides (mg/dL)	113 (82.60–149)	117 (90.64–139)	NS
TG:TCh	0.6 (0.46–0.80)	0.6 (0-50–0.80)	NS
TCh:HDL	7.0 (6.07–8.73)	7.5 (6.78–8.42)	NS
LDL:HDL	5.1 (4.05–6.55)	5.4 (4.90–6.42)	NS
ApoA (ng/mL)	2.5 (2.46–2.55)	2.5 (2.49–2.51)	NS
ApoB (ng/mL)	1.0 (0.98–1.04)	1.0 (0.96–1.04)	NS

Legend: BMI—body mass index; Hb—hemoglobin; Il-3—interleukin 3; TG—triglycerides; TCh—total cholesterol; ApoA—apolipoprotein A; ApoB—apolipoprotein B. NS—statistically insignificant.

**Table 2:** Erythrocyte antioxidant enzyme activity and glutathione concentration in the study groups.

	Lean n = 30 Median (Q_25_–Q_75_)	Overweight n = 27 Median (Q_25_–Q_75_)	*p<*
SOD (U/gHb)	762 (512–865)	872 (862–871)	0.02
CAT (U/gHb)	46 (29.66–57.80)	54 (49.11–59.61)	NS
GPX (U/gHb)	6.7 (5.67–7.68)	8.3 (5.85–9.89)	0.05
GST (U/gHb)	0.2 (0.10–0.25)	0.2 (0.10–0.21)	NS
GSSG-R (U/gHb)	0.1 (0.06–0.15)	0.1 (0.06–0.11)	NS
GSH_reduced_ (mM)	722 (667–749)	770 (716–787)	NS
GSSG (mM)	40 (27.45–47.81)	41 (36.40–47.65)	NS
GSH _total_ (mM)	762 (690–796)	811 (757–845)	NS
GSH:GSSG	17 (14.16–23.49)	19 (14.68–20.88)	NS

Legend: SOD—Superoxide dismutase; CAT—Catalase; GPX—Glutathione peroxidase; GST—Glutathione S-transferase; GSSG-R—Glutathione reductase; GSH—Glutathione; GSSG—Oxidized glutathione. NS—statistically insignificant.

## Data Availability

Not applicable.
